# A novel epigenetic marker, Ten-eleven translocation family member 2 (TET2), is identified in the intractable epileptic brain and regulates ATP binding cassette subfamily B member 1 (ABCB1) in the blood–brain barrier

**DOI:** 10.1080/21655979.2022.2045838

**Published:** 2022-03-02

**Authors:** Fan-Cheng Kong, Li-Qin Lang, Jie Hu, Xia-Ling Zhang, Ming-Kang Zhong, Chun-Lai Ma

**Affiliations:** a Department of Pharmacy, Huashan Hospital, Fudan University, Shanghai, China; b Department of Neurosurgery, Huashan Hospital at Fudan University, Shanghai, China; c Department of Pathology, Huashan Hospital, Fudan University, Shanghai, China

**Keywords:** Drug-resistant epilepsy, DNA methylation, TET2, ABCB1, blood-brain barrier

## Abstract

Drug-resistant epilepsy (DRE) is a chronic condition derived from spontaneous changes and regulatory effects in the epileptic brain. As demethylation factors, ten-eleven translocation (TET) family members have become a focus in recent studies of neurological disorders. Here, we quantified and localized TET1, TET2 and 5-hydroxymethylcytosine (5-hmC) in the temporal lobe cortex of DRE patients (n = 27) and traumatic brain hemorrhage controls (n = 10) by immunochemical staining. TET2 and ATP binding cassette subfamily B member 1 (ABCB1) expression patterns were determined in the isolated brain capillaries of DRE patients. TET2 expression was significantly increased in the temporal cortical tissue of DRE patients with or without hippocampal sclerosis (HS) compared to control patients, while TET1 and 5-hmC showed no differences in expression. We also found that a particularly strong expression of TET2 in the vascular tissue of DRE patients. ABCB1 and TET2 have evidently higher expression in the vascular endothelium from the neocortex of DRE patients. In blood–brain barrier (BBB) model, TET2 depletion can cause attenuated expression and function of ABCB1. Data from a cohort study and experiments in a BBB model suggest that TET2 has a specific regulatory effect on ABCB1, which may serve as a potential mechanism and target in DRE.

## Graphical Abstract

**Figure:**
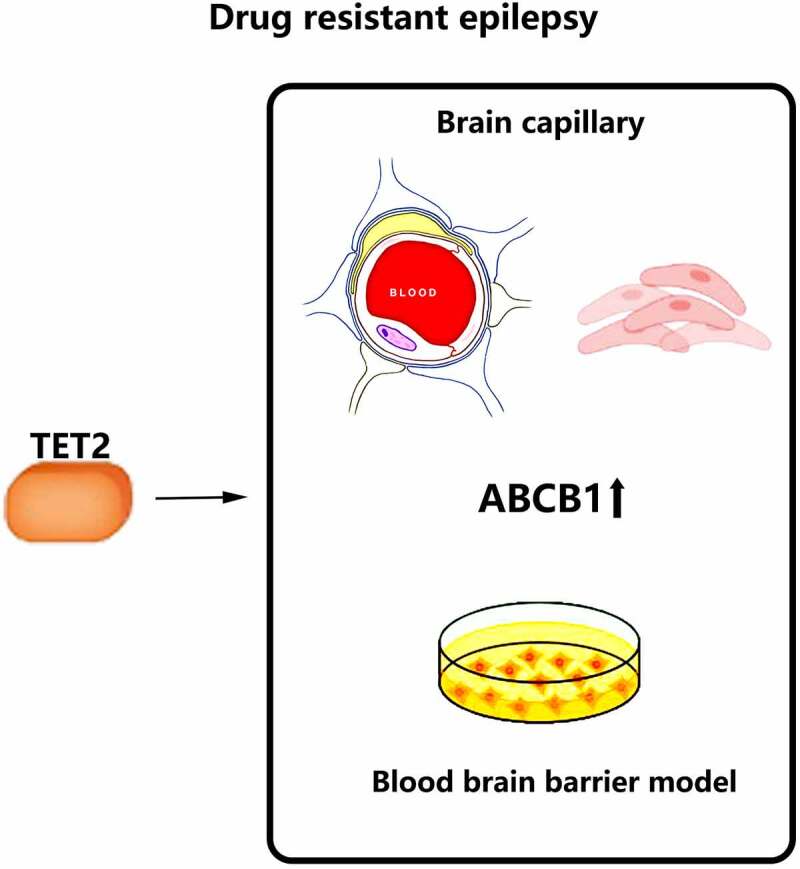

## 1. Introduction

The emergence of epigenetic modulators has revealed a promising future for the treatment of epilepsy. Although most patients can achieve ‘seizure-free’ status under the current drug therapy, 30% of these patients unavoidably develop medication resistance [1]. Hence, epigenetic markers seem to hold promise for ameliorating drug-resistance epilepsy in the next generation due to their flexibility and reversibility in a range of neurological disorders [2

Studies have reported epigenetic abnormalities involving DNA methylation, histone modification, and miRNA regulation in the epileptic brain [3–5]. Compared to alterations of histones and miRNAs, changes in DNA methylation may have a more durable and direct impact on gene expression [6]. As a catalytic element of CpG methylation enrichment, DNA methyltransferases (DNMTs) have the ability to induce DNA methylation, which mediates the gene silencing process by closing the binding area to transcription factors; they are suggested to be a contributing factor associated with epileptogenesis and recurrent seizure activity [7–9].

By contrast, demethylation by demethylases (DMs) exerts a robust activating effect on gene expression. Deregulation of DNA methylation has been directly linked to numerous epigenetic changes detected in biopsies from epilepsy patients [10–12] and seizure models [13,14]. Active DNA demethylation is carried out by ten-eleven translocation (TET) methylcytosine dioxygenases, which progressively oxidize 5-methylcytosine (5-mC) to 5-hydroxymethylcytosine (5-hmC), 5-formylcytosine (5-fC) and 5-carboxylcytosine (5-caC) [15,16]. Recently, TETs have been shown to play various roles in the physiology of neuroinflammation, neurodevelopment and memory formation[17–19]. Moreover, data from a transcriptome analysis of postoperative samples from patients with intractable epilepsy [20,21] indicated that TET1 and TET2 have unique mRNA splicing and expression patterns, respectively. Nonetheless, few studies have investigated the role of TET family members in epilepsy, especially the association of epigenetic changes with pivotal DRE-related alterations, to investigate the potential function of TET enzymes.

Based on transporter theory, pharmacoresistance in DRE may be caused by increased expression of multidrug efflux transporters in the cerebral vascular system, followed by insufficient penetration of antiepileptic drugs (AEDs) across the blood–brain barrier (BBB). P-glycoprotein (P-gp or ATP binding cassette subfamily B member 1, ABCB1) at the BBB is widely thought to restrict brain entry of multiple drugs [22,23]. Although the multifactorial causes of P-gp overexpression in epileptogenic brains with DRE are still unclear, the methylation mechanism may be involved [24], since fluctuating methylation levels of ABCB1 may be related to clinical consequences in a variety of multidrug-resistant conditions [25–27] in which the abundance of demethylases varies.

Considering the evidence discussed above, we hypothesized that TET enzymes might have a regulatory role in the expression of ABCB1 in drug-resistant epilepsy. In order to confirm our hypothesis, a pilot study was conducted to investigate the expression of TET1, TET2, and ABCB1 and the level of global methylation in the temporal cortex and vascular endothelium of patients with DRE and controls. And, we further explored the regulatory effect of the TET enzyme and the expression of ABCB1 in a BBB model simulated by cerebral endothelial cells.

## 2. Methods

### 2.1 Human epileptic brain specimens

Human brain tissue samples were obtained from 27 patients with drug-resistant TLE who were undergoing anterior temporal lobectomy. Patients were referred to the neurosurgical department of Huashan Hospital, Shanghai, for drug-resistant TLE. All patients were evaluated with ictal video-electroencephalography monitoring and magnetic resonance imaging with fluid-attenuated inversion recovery. The diagnoses of hippocampal sclerosis (HS) and non-sclerosis (non-HS) conformed to established diagnostic criteria [28]. For comparison, we obtained 10 neocortex specimens from patients treated for traumatic cerebral hemorrhage and 2 vascular resections from a patient with cerebral arteriovenous malformation. The specimens were taken only for therapeutic purposes. These controls had experienced a conventional neurological examination that revealed no signs of comorbidities or other central nervous diseases. They had no history of epilepsy or exposure to antiepileptic drugs.

This study was approved by the hospital Medical Ethics Committee (KY2019-607). All of the patients provided written informed consent for the use of surgical remnants. Patient and control demographics and pathologic diagnoses are presented in Tables 1 and 2. The drug regimen of epilepsy patients was recorded before surgery, and patients may have experienced other drug therapeutic strategy. The tissue for immunohistological detection was formalin-fixed, blocked in paraffin, and cut into 5 μm sections, which were then mounted onto slides. Tissues for WB were stored at −80°C after snap freezing.

**Table 1.** Clinical data of TLE patients

I.D.	Gender	Age (years)	Pathology	Antiepileptic drugs	Experimental use
P1	female	13	HS	OXC, LEV	IHC
P2	female	25	HS	OXC, LEV, GBP	IHC
P3	female	20	HS	LEV	IHC
P4	female	21	non-HS	OXC, CBZ, VPA	IHC
P5	female	24	non-HS	CBZ, LEV, VPA, TPM	IHC
P6	female	17	HS	OXC, LTG	IHC
P7	male	23	non-HS	LTG, TPM, CBZ	IHC
P8	female	35	non-HS	OXC	IHC
P9	female	52	HS	OXC, LTG	IHC
P10	male	37	non-HS	OXC	IHC
P11	male	21	HS	OXC	WB
P12	male	39	non-HS	CBZ, VPA	WB
P13	female	26	HS	OXC, LCM	WB
P14	female	29	HS	CBZ, TPM	WB
P15	female	34	HS	VPA	IHC, IF
P16	male	22	HS	VPA, LEV	IHC, IF
P17	male	31	non-HS	OXC	IHC, IF
P18	female	16	HS	OXC, LEV	IHC
P19	female	35	HS	LTG, OXC	IHC
P20	female	14	HS	CBZ	IHC
P21	female	60	HS	CBZ	IHC
P22	female	39	non-HS	LTG, LEV	IHC, IF
P23	female	17	non-HS	LEV, LTG, LCM	IHC
P24	female	30	HS	TPM, VPA	IHC
P25	female	43	non-HS	OXC	IHC
P26	male	18	HS	CBZ, LEV	IHC
P27	male	46	non-HS	LTG, VPA, TPM	IHC

HS, hippocampal sclerosis; non-HS, nonhippocampal sclerosis; OXC, oxcarbazepine; LEV, levetiracetam; GBP, gabapentin; VPA, valproate acid; TPM, topiramate; CBZ, carbamazepine; LTG, lamotrigine; LCM, lacosamide; IHC, immunochemistry; IF, immunofluorescence; WB, Western blot.

**Table 2.** Clinical data of controls

I.D.	Gender	Age (years)	Diagnosis	Experimental use
C1	female	63	traumatic cerebral hemorrhage	IHC
C2	male	52	traumatic cerebral hemorrhage	IHC
C3	female	66	traumatic cerebral hemorrhage	IHC
C4	male	54	traumatic cerebral hemorrhage	IHC
C5	male	48	traumatic cerebral hemorrhage	IHC
C6	male	65	traumatic cerebral hemorrhage	IHC
C7	male	47	traumatic cerebral hemorrhage	IHC
C8	female	55	traumatic cerebral hemorrhage	IHC
C9	female	44	traumatic cerebral hemorrhage	IHC
C10	male	61	traumatic cerebral hemorrhage	IHC
C11	male	42	cerebral arteriovenous malformation	WB
C12	male	48	cerebral arteriovenous malformation	WB

IHC, immunochemistry; WB, Western blot.

### 2.2 Immunohistochemistry

Slices were subjected to antigen retrieval in 0.01 M sodium citrate buffer (pH = 6) for 5 min with pressurization. After incubation in 3% H_2_O_2_-methanol for 10 min at room temperature, the slides were washed three times for 5 min in Phosphate Buffer (PBS). The sections were blocked for 30 min in normal goat serum (NGS) before incubation with primary antibodies against TET1 (1:6000, ab191698, Abcam), TET2 (1:30, ab243323, Abcam), and 5-hmC (1:800, ab214728, Abcam) diluted in TBS-T 0.3% NGS overnight at 4°C. The slides were washed three times for 5 min in PBS at room temperature. Then, the sections were incubated for 30 min at room temperature with secondary antibodies (JHBO1, Jiehao Biotechnology) diluted in TBS-T 0.3% NGS. After three washes in PBS, DAB-peroxidase substrate solution (pH 7.6) was used for chromogenic immunostaining for 5–10 min, and then the slides were washed for 15 min in water at room temperature. Slides were counterstained with hematoxylin for 5 minutes, dehydrated with gradient alcohol, cleared with xylene, and mounted with neutral gum. Ten fields of images were obtained using an PM 20 automatic microscope (Olympus, Tokyo, Japan).

### 2.3 Brain capillary isolation

Frozen brain capillaries were isolated as previously described [29,30]. Briefly, brains were harvested and collected in ice-cold DPBS containing Ca2+ and Mg2+, 5 mM d-glucose, and 1 mM sodium pyruvate, pH 7.4. The brain tissue was homogenized, mixed with Ficoll PM 400 (final concentration of 15%; Aladdin), and centrifuged (5800 g, 15 min, 4°C). The resulting capillary pellet was suspended in 1% BSA-DPBS and passed over 300 μm and 100 μm strainers in sequence, from which capillaries were collected in 1% BSA-DPBS. Then, the pellet was rinsed on a 100 μm filter with 1% BSA-DPBS. After collecting the capillaries, all samples were centrifuged two times at 1,500 g for 3 min at 4°C, and brain capillaries were used for Western blotting.

### 2.4 Western blotting

Isolated brain capillaries or hCMEC/D3 cells were homogenized in cold RIPA lysis buffer (Beyotime Biotechnology) containing phenylmethylsulfonyl fluoride. Samples were mixed with 5× lithium dodecyl sulfate sample buffer and 10% DTT reducing agent. The samples were run in 4–20% Tris-Gly gradient gels and transferred to the eBlot® L1 Fast Wet Transfer System (Genscript, USA). Membranes were blocked for 1 h and incubated overnight at 4°C with primary antibodies against GAPDH (1:5000, ab8226, Abcam), TET2 (1:500, #18,950, Cell Signaling), or P-gp (1:1000, ab170904, Abcam). The membranes were washed and incubated with horseradish peroxidase–conjugated secondary IgG (1:5000; SGARHAP, Yishan Biotech) for 1 h at RT. Protein bands were visualized using a BeyoECL Star Kit (Beyotime) and an ImageQuant LAS 4000 luminometer (GE, USA). The optical density of the protein bands was measured with ImageJ software (NIH, Bethesda, MD, USA).

### 2.5 Cell culture

Human brain capillary endothelial cells (hCMEC/D3) were purchased from Fu Heng Biology (Shanghai) and grown in Endothelial Cell Medium (ECM) (1001, ScienCell) supplemented with 5% characterized fetal bovine serum (0025, ScienCell), 1 ng/mL basic fibroblast growth factor (1052, ScienCell), and 1% penicillin-streptomycin (0503, ScienCell). Cells were cultured at 37°C in a humidified incubator with 5% CO_2_.

### 2.6 siRNA transfection

Gene‐specific human TET2 small interfering RNA (siRNA) oligonucleotides were synthesized by RiboBio and used at a final concentration of 1 μm for hCMEC/D3 in the respective experiments. Non-silencing control siRNAs were constructed by RiboBio and the target sequences of the siRNAs are provided in the Supplemental Materials. Cells were seeded in 6-well plates at 70% confluence and transiently transfected with TET2 siRNA (50 nM) and the corresponding silencing negative control (siNC) using Lipofectamine RNAiMAX Reagent (Thermo Fisher Scientific). Experiments were performed 24 h or 48 h after transfection.

### 2.7 RT-qPCR

Total RNA was isolated from the cells using Trizol reagent (Invitrogen, USA). Purity and concentration of total RNA were measured by a Nanodrop 1000 spectrophotometer (Thermo Scientific, Rockford, IL). Reverse transcription was performed by FastKing gDNA Dispelling RT SuperMix with RNase Inhibitor (TIANGEN, Beijing). Specific forward and reverse primers (see Supplementary Materials) for each gene were constructed by BioTNT Company. Then, mRNA expression was detected using SuperReal PreMix Plus Kit (Tiangen, Beijing). Reaction was performed in a 96-well plate format using an ABI™7500 real-time PCR instrument (Applied Biosystems). Ct values were converted to comparative Ct values (2^−ΔΔCt^) by comparison to reference gene GAPDH.

### 2.8 Rhodamine 123 accumulation assay

P-gp-mediated efflux was measured by detecting the intracellular fluorescence of rhodamine 123 (Rho123) via flow cytometry as previously described [31]. Briefly, approximately 5 × 10^5^ cells were collected and incubated in 1 ml cell medium with 2 μmol/L Rho123 for 30 min at 37°C. Then, Rho123 dye was removed by centrifugation and washed cells twice with PBS. Resuspended endothelial cells and incubated them in cell medium for 1 h. Negative control were cells without Rho123 dye. The fluorescence of intracellular Rho123 was calculated using a BD FACS Canton flow cytometer (Beckman) (excitation/emission wavelength: 485/530 nm).

### 2.9 Statistical analysis

GraphPad Prism v9© (La Jolla, CA) and SPSS v22.0 (IBM, USA) software were used for figure creation and statistical analysis, respectively. Significant differences between groups were assessed using Student’s t-test and one-way analysis of variance (ANOVA) as appropriate for comparations. Statistical significance was set at p < 0.05.

## 3. Results

This study aims to demonstrate the significant expression of TET2 in the cerebrovascular tissue of drug-resistant epilepsy. Additionally, expression and functionality of ABCB1 were detected in hCMECs/D3 after silencing TET2 in order to reveal the regulation effects of ABCB1 by TET2 in blood–brain barrier model.

### 3.1 Expression of the TET proteins in temporal cortex of patients with DRE

**Figure 1.**
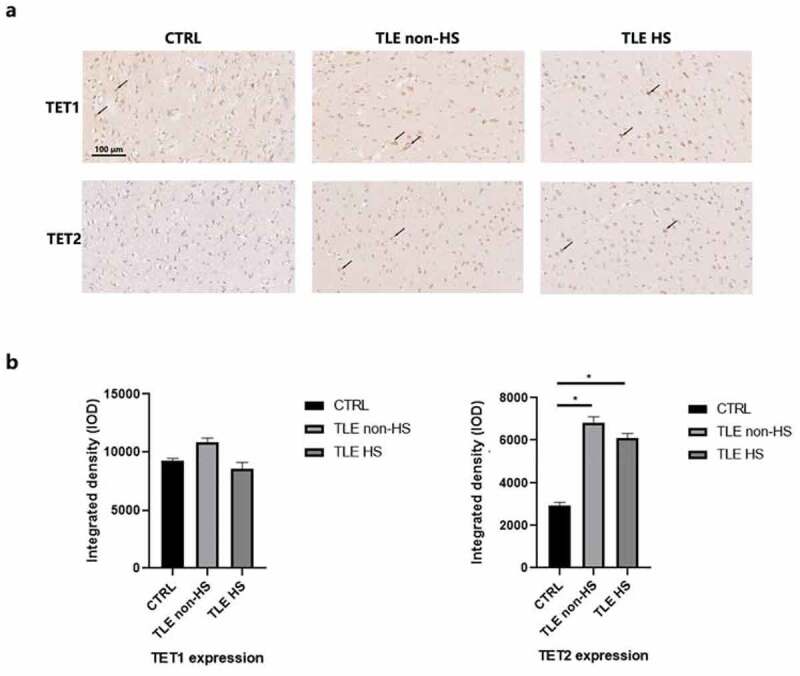
**Immunoreactivity of TET1, TET2 and ABCB1 in the neocortex of TLE patients.** a. Representative image of TET1 and TET2 staining of the temporal cortex of TLE patients divided into HS (n = 16), non-HS (n = 11) and control (n = 10) groups. Scale bars are equal to 100 μm. Arrow heads indicate the positive cells. b. Quantitative analysis of the integrated density of TET1 and TET2 reactivity. *p < 0.05 indicates asignificant difference from CTRL. TLE, temporal lobe epilepsy; TLE HS, TLE patients with hippocampal sclerosis; TLE non-HS, TLE patients without hippocampal sclerosis; CTRL, control.

To observe the expression of the TET protein, DRE patients were divided into hippocampal sclerosis (n = 16) and nonhippocampal sclerosis (n = 11) groups. We first located TET1 and TET2 in the temporal neocortex of 10 controls and 27 TLE patients using IHC staining (Figure 1a). TET1 and TET2 are extensively labeled in the cytoplasm and nucleus of neurons, astrocytes, and endothelial cells at the temporal cortex interface. Semiquantitative analysis of staining (Figure 1b) demonstrated a significant increase in TET2 in DRE patients compared to controls. TET1 had high immunopositivity in both patients and controls, but their quantitative results showed no significant difference.

       

### 3.2 Expression of TET2 and ABCB1 in vascular endothelial cells of patients with DRE

**Figure 2.**
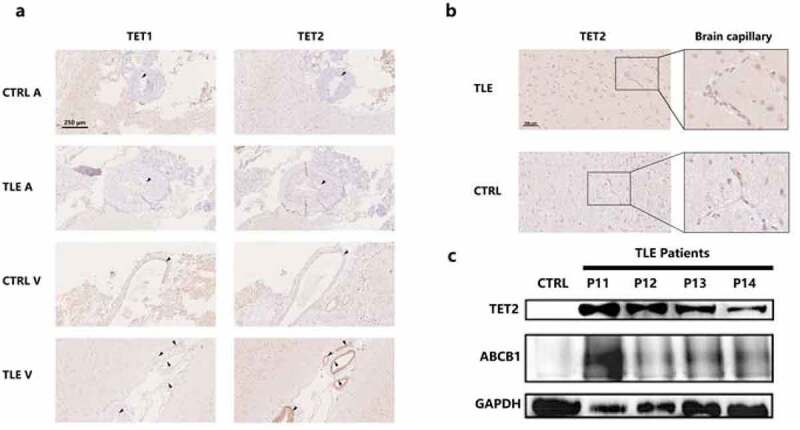
**Differential involvement of TET1, TET2, and ABCB1 in the cerebral vasculature of the neocortex.** a. Expression patterns of TET1 and TET2 in the veins and arteries of the temporal cortex in TLE patients and controls. Arrow heads indicate the endothelial structure of the cerebral vasculature. Scale bars are equal to 250 μm. b. TET2 staining in brain capillaries with amplification of the temporal cortex of TLE patients and controls. Scale bars are equal to 100 μm. c. Western blot showing TET2 and ABCB1 expression in isolated brain capillaries of TLE patients (P11-P14) and controls. V, vein; A, artery; TLE, temporal lobe epilepsy; CTRL, control.

Dysfunction of the BBB and overexpression of ABCB1 in brain capillaries induce regional metabolic abnormalities [32], thereby attenuating the penetration of AEDs. Moreover, methylation factors may have an impact on this procedure. To precisely determine the expression patterns of the TET protein in the vascular structure, we solely investigated the arteries and veins in the temporal cortex of DRE patients and controls (Figure 2a) and directly observed the expression distribution of TET1 and TET2. Astonishingly, arrowheads specifically marking the endothelial section showed that TET2 had strong immunopositivity in the endothelium, especially in the venae meningeal. However, in both epilepsy patients and controls, TET1 showed a relatively weak immunosignal in the endothelium. Moreover, no positive TET1 and TET2 signals were detected in vascular smooth muscle. Brain capillaries show particularly fine TET2-positive staining in cytoplasm (Figure 2b).

According to previously reported methods [29,30], we isolated and purified the vascular endothelium from DRE patients (n = 4, P11-P14) and controls to perform TET2 and ABCB1 quantitative analysis by WB (Figure 2c). Since resection samples of brains with traumatic brain hemorrhage contain a mass of blood clots that cannot be completely dislodged, we collected vascular biopsies from patients with cerebral arteriovenous malformations as a control. Although there are differences in the expression of TET2 and ABCB1 among individual DRE patients, both TET2 and ABCB1 have abundant expression in the vascular endothelium of DRE patients compared to controls.

### 3.3 5-hmC levels in the cortex of patients with drug-resistant TLE

**Figure 3.**
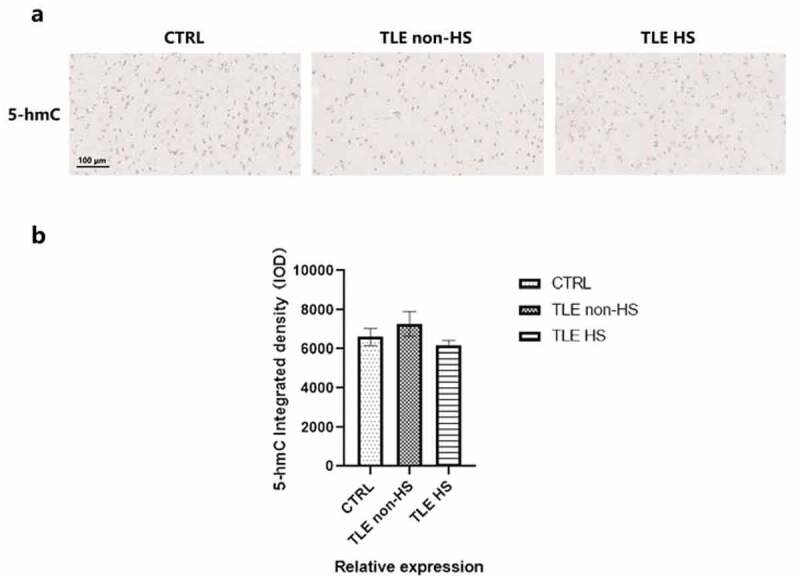
**Global 5-hmC patterns in the temporal cortex of TLE patients and controls.** a. Representative image of 5-hmC staining in TLE patients divided into HS (n = 16), non-HS (n = 11) and control (n = 10) groups. b. Quantitative analysis of the integrated density of 5-hmC in the neocortex. *p < 0.05 indicates asignificant difference from CTRL. TLE, temporal lobe epilepsy; TLE HS, TLE patients with hippocampal sclerosis; TLE non-HS, TLE patients without hippocampal sclerosis; CTRL, control.

TET enzymes can catalyze the demethylation activity from 5-mC to 5-hmC, and 5-hmC enrichment can be reflective of the global methylation level and hydroxymethylation. To further evaluate the demethylation changes mediated by the TET protein, 5-hmC was detected in the temporal cortical tissue of DRE patients, divided into HS (n = 16), non-HS (n = 11) and control (n = 10) groups (Figure 3a). Semiquantitative image analysis was used to quantify the integrated density of 5-hmC between the drug-resistant TLE patients and controls (Figure 3b). However, the 5-hmC integrated density was not significantly decreased or elevated in all patient groups compared to the controls, which means that it is possible that the demethylation effect produced by the difference in the expression of the two TET proteins has little effect on the global methylation level.

      

### 3.4 The expression and function of ABCB1 after TET2 depletion

The hCMEC/D3 cell line was derived from human temporal lobe microvessels isolated from tissue excised during surgery for the control of epilepsy. An adequate pattern of transporter expression makes it a suitable in vitro human BBB model [33]. In our preliminary experiments, we discovered that TET2 and ABCB1 can be stably expressed in the normal hCMEC/D3 cell line. Therefore, we attempted to knock down TET2 by siRNA transfection to explore the regulatory effects between TET2 and ABCB1 in hCMEC/D3 cells.

**Figure 4.**
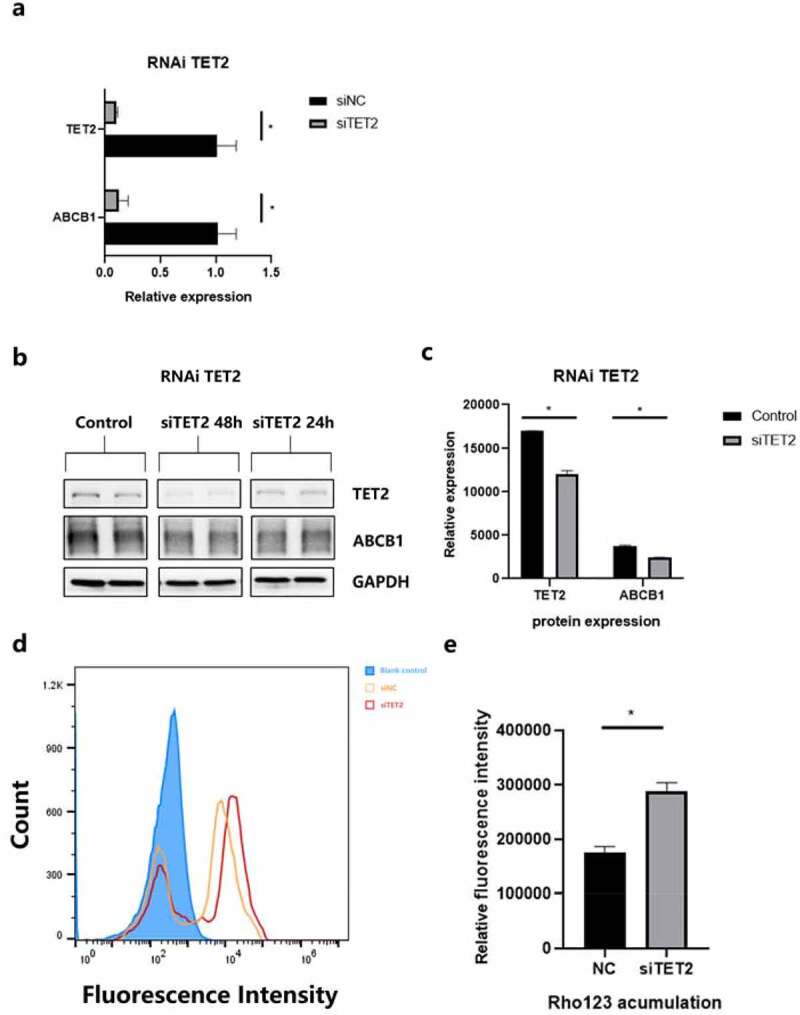
**TET2 regulates ABCB1 expression and function in hCMEC/D3 cells.** a.qPCR data showing the relative expression of TET2 and ABCB1 after siRNA transfection of TET2. *p < 0.05 indicates asignificant difference from siNC. b.Western blot showing TET2 and ABCB1 expression after TET2 depletion at 24 h and 48 h, respectively. c.Quantification of Western blot bands to determine TET2 and ABCB1 expression after TET2 depletion. *p < 0.05 indicates asignificant difference from siNC. d.Intracellular accumulation of Rho123 in hCMEC/D3 cells was determined by flow cytometry. e.Quantification of Rho123 uptake after TET2 depletion. *p < 0.05 indicates asignificant difference from siNC. siTET2, silencing TET2; siNC, silencing negative control.

Quantitative PCR was used to tentatively increase the transfection efficiency and variation tendency of ABCB1 (Figure 4a). The results showed that TET2 depletion can cause a significant decrease in ABCB1. Subsequently, WB revealed the changes in the protein levels of ABCB1 and TET2 at 24 h and 48 h after TET2 depletion (Figure 4b). Quantification of the bands by image analysis showed that as siTET2 treatment was performed, the expression of ABCB1 was significantly reduced (Figure 4c), indicating that TET2 can regulate the change in ABCB1 expression.

Rho 123 is an idiosyncratic substrate of P-gp and is thus widely used in the efflux functionality of transporters [34]. The accumulation of Rho123 in efflux phase fluorescence detected by flow cytometry can represent the efflux function of ABCB1 in hCMEC/D3 cells. Figure 4d shows the results of flow fluorescence detection of Rho123. A histogram (Figure 4e) showed that cells after siTET2 treatment had an evident residue of Rho123 compared to the interference negative control (siNC), indicating a weaker ABCB1 efflux function, which is also consistent with the ABCB1 expression results described in the previous section. In conclusion, the reduction in TET2 levels also causes the overall ABCB1 efflux function to decline.

 

## 4. Discussion

Our research integrating epigenetic modulators and multidrug resistance factors with the analysis of DRE patients and BBB model systems provides insight into the possible molecular mechanisms involved in DRE.

As chromosomal translocation partners, TET family members, including TET1 and TET2, were initially found in leukemia and proven to be key regulators of DNA demethylation owing to their dioxygenase activity [35]. Recently, numerous studies have indicated the significance of TET2 proteins and 5-hmC in epigenetic regulation in neurodegenerative conditions such as Parkinson’s disease and Alzheimer’s disease [36–38]. Similar to previous approaches searching for methylated markers, such as DNMT [8], Reelin [39] and brain derived neurotrophic factor [40], we first observed the expression and location of TET1 and TET2 in the HS and non-HS temporal neocortex in DRE, which resulted in the significant discovery that TET2 expression is extensively induced in focal lesions of DRE. 5-hmC is the most stable and abundant product of TET enzymatic activity, which can be well recognized by the fact that the active TET methylation machinery correlates with chromatin accessibility [41]. Our result of 5-hmC in the DRE neocortex was in line with de Nijs, L. et al.’s finding [8] and did not show specific changes in DRE patients compared with controls. We think there may be other demethylation pathways that offset the corresponding effects or cannot be evaluated merely by the 5-hmC level. For example, a recent study indicated that the fC/caC pathway promotes rapid DNA demethylation at reprogramming loci, even though 5-hmC is maintained at a steady-state abundance [42].

In addition, TET2’s role in the innate immune response allows it to function in a large number of pathophysiological processes associated with inflammatory diseases, which establish a ‘bridge’ connecting it to cerebrovascular inflammation [43]. However, whether it exerts protective or deteriorative effects in various diseases appears entirely distinct, e.g., decreased TET2 expression may exacerbate vasculitis and adverse vascular remodeling of pulmonary arterial hypertension [44], while elevated TET2 expression causes neuronal damage and loss [36]. Advances in our understanding of the mechanisms that govern neuroinflammation in epilepsy, particularly in the BBB, also raised some considerations concerning its importance in the clinical management of seizures [45]. Neuroinflammation can provoke BBB dysfunction and P-gp induction in seizure models, which focus on COX-2 [46–48] and IL-1β [49] signals in vascular endothelial cells. Further BBB disruption might have functional effects on the therapeutic effects of antiepileptic drugs, thereby causing DRE. This is why we shifted our attention to the transporter thesis. Consequently, to investigate the potential association between TET2 and P-gp, we tentatively examined the expression status in histologic samples and isolated cerebral vessels of the DRE temporal lobe. Both TET2 and P-gp had higher expression than the control. In a BBB-simulated model constructed with hCMEC/D3 cells, TET2 depletion led to a reduction in the transcription, protein expression, and efflux function of P-gp, which suggested that TET2 has a positive regulatory impact on P-gp in the brain endothelium. And our analysis method of P-gp function is prevalently used for verification of tumor resistant [50].

Our research also has limitations related to sample size and the failure to explore specific mechanisms, such as inflammatory pathways, in the seizure model. In addition, we did not study astrocytes, an important component of the BBB. Because firstly, previous studies have discovered that P-gp mainly concentrate in vascular endothelial cells of epileptic brains [23]. Second, a more ideal and reasonable blood–brain barrier models like neurovascular unit are still in the process of testing [51].

Interestingly, N-methyl D-aspartate antagonists and COX-2 inhibitors, such as celecoxib, have been shown to prevent the seizure-induced increase in P-gp functionality, thereby reversing AED resistance in rats [52]. The rapidly increase of glutamate stimulants in seizure in vitro and in vivo models and overactivation of glutamate receptors were considered to be an important trigger for the increase of P-gp in brain capillary endothelial cells. Thus, the chronic epilepsy model exposed to glutamate seem to be a reasonable option to investigate the latent inflammatory framework of TET2 and its relationship with pharmacoresistance and BBB dysfunction [53–55].

## 5 Conclusion

In summary, the results of this study indicate a significant expression of TET2 in pharmacoresistant epilepticus brain, which support TET2 as a possible epigenetic marker in DRE. In addition, the manipulation of P-gp expression and functionality by TET2 in a BBB model underlies its involvement in the progression of pharmacoresistant epilepsy. The next steps for research in future studies could focus on identifying inflammatory mechanisms in combination with epigenetic targeting of TET2 in epileptogenesis and structural alterations of BBB.

## Data Availability

The original data is available from the corresponding authors on reasonable request.
